# Does matching degree matter for proximal femoral intramedullary nail on reoperation rate in intertrochanteric fractures?

**DOI:** 10.1186/s13018-022-03476-9

**Published:** 2022-12-29

**Authors:** Fei Wang, Ji-Long Zou, Jian Shang

**Affiliations:** 1 The First Affiliated Hospital of Harbin Medical University, No. 23 Post Office Street, Nangang District, Harbin, Heilongjiang China; 2 Shenzhen University General Hospital, Shenzhen University, 1098 Xueyuan Avenue, Xili University Town, Nanshan District, Shenzhen, Guangdong China

**Keywords:** Intertrochanteric fracture, Matching degree, Gap, Reoperation

## Abstract

**Introduction:**

Previous articles reported on the tip–apex distance, lag screw placement, fracture pattern, reduction quality, osteoporosis and other factors associated with second surgery. The current study focused on investigating the association of the matching degree between proximal femoral intramedullary nail and femoral medullary cavity on reoperation rate.

**Patients and methods:**

A retrospective cohort study was conducted. It included patients with intertrochanteric fracture who were treated with proximal femoral anti-rotatory intramedullary nail (PFNA) between January 2016 and April 2021. The gap between the intramedullary nail and the femoral medullary cavity was equal to the difference in diameter between the two. According to the gap size, all patients were divided into three groups, as follows: high-matching group: gap ≤ 2 mm; middle-matching group: 2 < gap < 4 mm; and low-matching group: gap ≥ 4 mm. The mean gap was measured through standard images. The primary observational index was whether the reoperation was needed, and secondary observational indexes included operative time, length of hospital stay. Patient characteristics were recorded, as follows: age, sex, follow-up time, fracture pattern, reduction grade and length of intramedullary nail.

**Results:**

A total of 203 eligible patients were recorded, including 78 males (38.4%) and 125 females (61.6%). They had a mean age of 77.8 ± 9.9 years old and an average follow-up time of 58.1 ± 24.0 weeks. Twenty-seven patients (13.3%) needed a second operation. Coxa varus combined with screw cutting was the most common reason for reoperation (11 cases). Unstable fracture pattern with poor reduction grade tended to contribute to reoperation, whose odds ratio (OR) was 6.61 (95% confidence interval [CI], 1.98–22.09; *P* = 0.002). The three groups had 11 cases (13.7%), 12 cases (13.8%) and 4 cases (11.1%) of reoperation, respectively, and logistic regression showed no significant association was noted between matching degree of intramedullary nail and reoperation rate.

**Conclusions:**

The matching degree between proximal femoral intramedullary nail and femoral medullary cavity did not seem to be an important factor for reoperation, which offered more options of intramedullary nail size intraoperatively and reduced implants stock from inventory.

## Introduction

Intertrochanteric fracture occurs in elderly commonly, and most patients lose part of the hip function, which brings great burden to their families and constitutes a large part of the healthcare burden [1, 2]. Intramedullary nailing has become a common treatment for intertrochanteric fracture. It is applied to various types of fracture with a good biomechanical performance. Intramedullary nailing allows patients to walk immediately after surgery [3, 4]. But implant failures are often reported. Previous studies have reported the failure patterns of internal fixation, including coxa varus, screw cutting, screw backing-out, prosthesis peripheral fracture and non-union [5–7]. Some studies forecasted the risk factors of implant failures, such as the tip–apex distance, lag screw placement, fracture pattern, reduction grade and osteoporosis [8–13]. However, few studies were related to the effect of the matching degree between proximal femoral intramedullary nail and femoral medullary cavity on reoperation rate. The current study focuses on this effect to serve as a guide for choosing the size of nail intraoperatively.

## Patients and methods

**Fig. 1:**
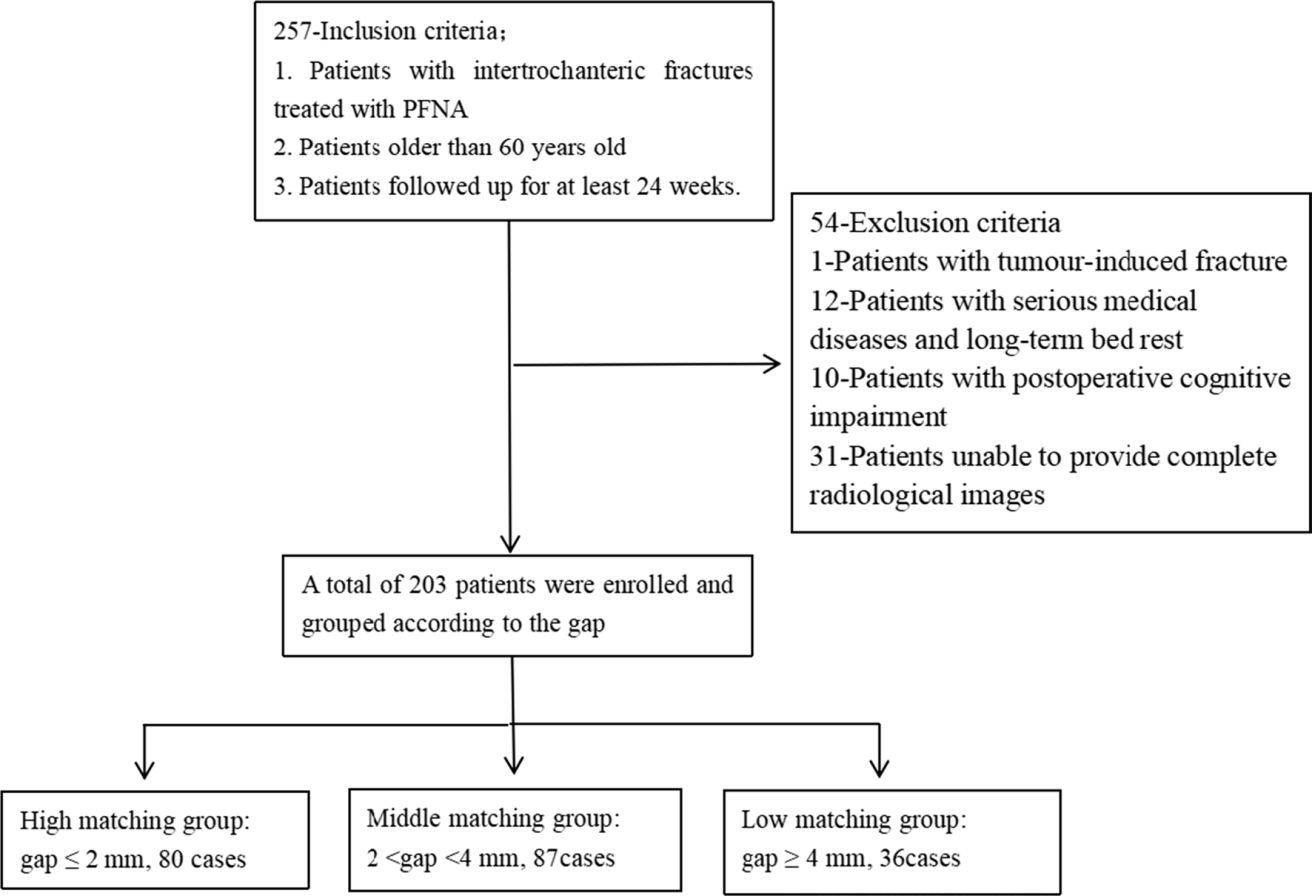
Patient screening flowchart

After obtaining the approval of the Ethics Committee of the First Affiliated Hospital of Harbin Medical University, a retrospective cohort study was conducted on patients with intertrochanteric fracture who were treated with proximal femoral anti-rotatory intramedullary nail (PFNA) between January 2016 and April 2021. Inclusion criteria included patients who were older than 60 years old and followed up for at least 24 weeks. Exclusion criteria included patients who had tumor-induced fracture, had serious medical disease and had to undergo long-term bed rest had postoperative cognitive impairment and were unable to provide complete radiological image. The primary observational index was whether the reoperation was needed, and secondary observational indexes included operative time and length of hospital stay. Patient characteristics were recorded, as follows: age, sex, follow-up time, fracture pattern, reduction grade and length of intramedullary nail. The causes of reoperation were recorded, including coxa varus, screw cutting (extrusion with no cranial perforation), screw cutting-out (a cranial perforation), screw backing-out, prosthesis peripheral fracture, non-union and complications associated with internal fixation (Fig. 1).

Fractures were classified according to OTA/AO classification [14]. OTA/AO 31A1 fractures were defined as stable fractures, and 31A2 and 31A3 fractures were defined as unstable fractures [15]. According to the experience of Baumgaertner et al., the standard reduction was defined as follows. (1) The distance of the fracture fragment was < 4 mm on standard anteroposterior and lateral radiographs. (2) The neck–shaft angle on AP view was normal or slightly valved (130°–150°), and the angulation of fracture fragment was < 20° on Lat view. If both criteria were met, then the reduction quality was classified as good. If one criterion was met, then the reduction quality was classified as acceptable. If neither criteria were met, then it was classified as poor [16]. There were three types of intramedullary nail lengths used, namely 170, 200 and 240 mm. Only one distal static locking bolt was secured to the intramedullary nail due to the habit of the surgeon. The operation was performed by different surgeons who were given the same training on the surgical approach, posture management and pre- and postoperative management to decrease errors.

**Fig. 2:**
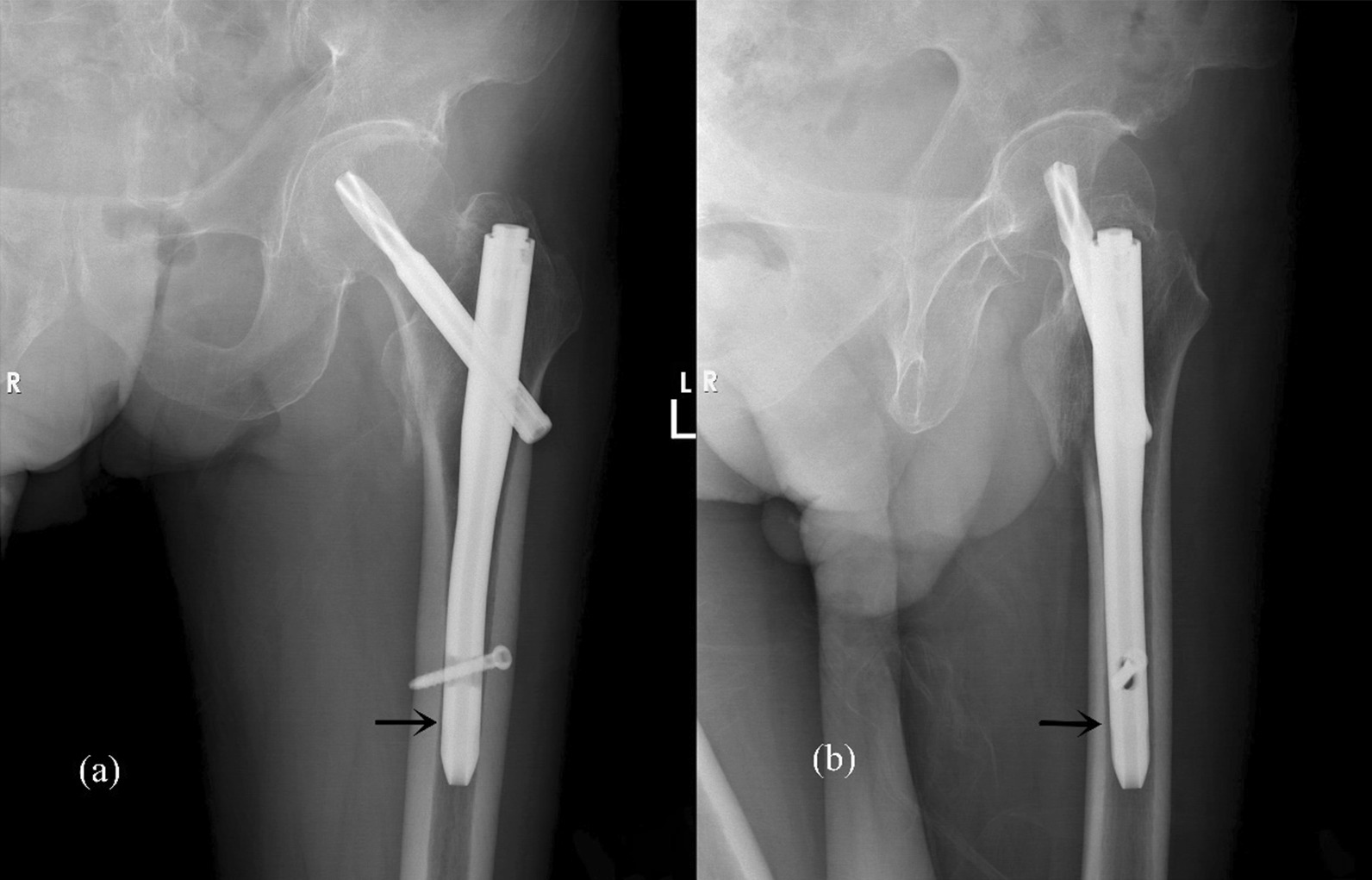
**a** AP radiograph. **b** Lateral radiograph. The diameter of femoral medullary cavity was measured at the layer where the medullary cavity was most fully filled with the distal nail.

To evaluate whether patients have achieved bone union, three criteria must be met, as follows: (1) at least three cortices showing continuous callus formation on standard anteroposterior and lateral radiographs; (2) absence of obvious pain on palpation and percussion at original fracture site; and (3) ability to walk without auxiliary devices [17, 18]. The length and diameter of the intramedullary nail can be obtained from the surgical record. Considering that the diameter of femoral medullary cavity was not uniform, the diameter was measured at the level where the medullary cavity is most fully filled with distal nail by ImedPacs software system (Dong Hua Software Company, China) (Fig. 2). The gap between the intramedullary nail and the femoral medullary cavity was equal to the difference in diameter between the two, and all patients were divided into three groups according to the gap: high-matching group: gap ≤ 2 mm; middle-matching group: 2 < gap < 4 mm; and low-matching group: gap ≥ 4 mm. The grouping method was based on the experience of Richard et al. [19] and the actual situation of this cohort study.

### Statistical analysis

The statistical analysis was performed using R version 4.0.3. A chi-square test was used to compare homogeneous distribution among categorical variables by evaluating frequencies within the groups. A one-way analysis of variance (ANOVA) test was used for comparing homogeneous distribution among numerical variables. Logistic regression was performed to analyze the relationships of reoperation with age, sex, fracture pattern and reduction quality, nail length and matching degree.

## Result

A total of 203 patients were finally enrolled (Table 1). Eighty patients were in high-matching group (gap ≤ 2 mm), 87 cases were in middle-matching group (2 mm < gap < 4 mm) and 36 patients were in low-matching group (gap ≥ 4 mm). There were 78 males (38.4%) and 125 females (61.6%). The mean age of the patients was 77.8 ± 9.9 years old, and the mean follow-up time was 58.1 ± 24.0 weeks. Forty-eight cases were considered as stable fracture (23.6%), and 155 cases were considered as unstable fracture (76.4%). The cases with good, acceptable and poor fracture reduction grades were 103 (50.7%), 67 (33.0%) and 33 (16.3%), respectively.

**Table 1:** Characteristics of intertrochanteric fractures in three groups

Characteristic	High-matching group	Middle-matching group	Low-matching group	Combination
Patient, *n* (%)	80 (39.4%)	87 (42.9%)	36 (17.7%)	203 (100%)
Age, mean ± SD (year)	76.6 ± 10.7	78.8 ± 9.3	78.1 ± 9.8	77.8 ± 9.9
Gender, *n* (%)				
Male	37 (46.3%)	32 (36.8%)	9 (25.0%)	78 (38.4%)
Female	43 (53.7%)	55 (63.2%)	27 (75.0%)	125 (61.6%)
Follow-up time, mean ± SD (week)	58.8 ± 23.9	58.9 ± 24.1	54.7 ± 24.7	58.1 ± 24.0
Length of stay, mean ± SD (day)	9.8 ± 4.3	9.3 ± 4.0	8.6 ± 3.6	9.4 ± 4.1
Operating time, mean ± SD (min)	120.7 ± 43.2	107.7 ± 35.2	121.2 ± 43.4	115.2 ± 40.3
Fracture, *n* (%)				
Stable	21 (26.3%)	19 (21.8%)	8 (22.2%)	48 (23.6%)
Unstable	59 (73.7%)	68 (78.2%)	28 (77.8%)	155 (76.4%)
Quality of reduction, *n* (%)				
Good	41 (51.2%)	43 (49.4%)	19 (52.8%)	103 (50.7%)
Acceptable	26 (32.5%)	33 (37.9%)	8 (22.2%)	67 (33.0%)
Poor	13 (16.3%)	11 (12.7%)	9 (25.0%)	33 (16.3%)
Nail type, *n* (%)				
170 mm	27 (33.7%)	20 (23.0%)	9 (25.0%)	56 (27.6%)
200 mm	45 (56.3%)	64 (73.6%)	27 (75.0%)	136 (67.0%)
240 mm	8 (10.0%)	3 (3.4%)	0 (0%)	11 (5.4%)
Postoperative outcome, *n* (%)				
Non-reoperation	69 (86.3%)	75 (86.2%)	32 (88.9%)	176 (86.7%)
Reoperation	11 (13.7%)	12 (13.8%)	4 (11.1%)	27 (13.3%)

Twenty-seven patients (13.3%) required additional surgery. The most common cause of reoperation is coxa varus combined with cutting of screw (11 cases). Other causes of reoperation include coxa varus combined with cutting-out of screw (two cases), coxa varus combined with back nail (three cases), simple coxa varus (two cases); non-union (four cases), pain associated with intramedullary nail stimulation (five cases). The three groups had 11 cases (13.7%), 12 cases (13.8%) and 4 cases (11.1%) of reoperation, respectively, and no statistically significant difference was found among the groups (*P* = 0.925).

Statistically significant differences were not observed among the three groups in age, gender, follow-up time, length of hospital stay, fracture patterns and reduction quality, as shown in Table 2. However, a significant difference was found in the length of intramedullary nail (*P* = 0.049). The differences of operative time among the three groups were also analyzed, and significant differences were not observed (*P* = 0.071, 0.681, 0.767).

**Table 2:** Statistically correlated results of three groups

Characteristic	High-matching group	Middle-matching group	Low-matching group	*P* value
Age, mean ± SD (year)	76.6 ± 10.7	78.8 ± 9.3	78.1 ± 9.8	0.352
Female gender, *n* (%)	43 (53.7%)	55 (63.2%)	27 (75.0%)	0.086
Follow-up time, mean ± SD (week)	58.8 ± 23.9	58.9 ± 24.1	54.7 ± 24.7	0.642
Length of stay, mean ± SD (day)	9.8 ± 4.3	9.3 ± 4.0	8.6 ± 3.6	0.342
Operating time, mean ± SD (min)	120.7 ± 43.2	107.7 ± 35.2	121.2 ± 43.4	0.071
Unstable fracture pattern, n (%)	59 (73.7%)	68 (78.2%)	28 (77.8%)	0.779
Poor reduction grade, *n* (%)	13 (16.3%)	11 (12.7%)	9 (25.0%)	0.358
240 mm nail, *n* (%)	8 (10.0%)	3 (3.4%)	0 (0%)	0.049
Reoperation, *n* (%)	11 (13.7%)	12 (13.8%)	4 (11.1%)	0.925

Logistic regression analysis was conducted (Table 3). Age, sex, nail length and gap size were not significant. Therefore, no significant association was found among them and reoperation. No strong dependency was observed among explanatory variables, except between reduction quality and fracture pattern (6.798e−13). Therefore, these two variables were combined together to one categorical variable with five levels (Stable, good; Stable, acceptable; Unstable, good; Unstable, acceptable; and Unstable, poor). Unstable fracture pattern with poor reduction grade tended to contribute to reoperation, whose odds ratio (OR) was 6.61 (95% confidence interval [CI], 1.98–22.09; *P* = 0.002). Result showed matching degree was not an important factor of reoperation.

**Table 3:** Logistic regression analysis

Regression model	Odds ratio	95% Confidence interval	*P* value
Age	1.01	0.96–1.05	0.811
Gender			
Male	0.42	0.15–1.20	0.106
Female	Ref	Ref	Ref
Fracture pattern and reduction grade			
Stable, good	0.19	0.02–1.62	0.129
Stable, acceptable	1.14	0.12–11.19	0.909
Unstable, good	0.77	0.22–2.67	0.683
Unstable, acceptable	Ref	Ref	Ref
Unstable, poor	6.61	1.98–22.09	0.002
Nail length			
170 mm	Ref	Ref	Ref
200 mm	1.21	0.39–3.77	0.739
240 mm	2.63	0.45–15.41	0.284
Matching degree			
High-matching degree	Ref	Ref	Ref
Middle-matching degree	2.43	0.58–10.23	0.225
Low-matching degree	2.22	0.50–9.94	0.295

## Discussion

The matching degree between the nail and femoral medullary cavity might affect fracture healing [20]. Millar et al.’s study of femoral shaft fractures asserted that a satisfactory nail fit allows smaller interfragmentary movement, which results in a more satisfactory outcome; they recommended an ideal nail fit of 90% at the isthmus to avoid surgical re-intervention [20]. However, the biomechanics of intertrochanteric fractures are different from those of femoral shaft fractures. Studies describing matching rate as an indicator of fracture healing and stability for intertrochanteric fractures are few. Some scholars have compared the effect of the matching degree of intramedullary nail on stability from the perspective of biomechanics. Simpson et al. conducted a finite element analysis of the nail, which showed that the stability and stiffness of the implant bone decreased and the von Mises stress in the nail and bone increased with the decrease in the matching rate between the nail and the femoral bone marrow cavity [21]. A biomechanical study of 70 composite femur models by Durusoy et al. revealed that large-diameter intramedullary nail increases diaphyseal adherence to reduce the movement of the intramedullary nail in the femoral medullary cavity and decrease the risk of varus collapse and cutting rate of the screw, consequently [22]. Previous works demonstrated the higher matching degree of the nail might offer more satisfactory biomechanical properties for the nail–bone system.

Whether a nail–bone system can meet the need for fracture healing depends on a variety of factors, not merely biomechanics [23]. More clinical analyses are needed to define the effect of the nail–canal gap on fracture healing. Subsequent clinical studies assessed the reoperation rate of proximal femoral intramedullary nails with diameters of 10 and > 10 mm in the treatment of intertrochanteric fractures and found no significant difference between the two groups (*P* > 0.05) [15]; however, this study did not take into account the effect of individual differences in femoral marrow cavity. The matching degree of the nail depends on the difference or ratio of the diameter of the femoral marrow cavity and that of the intramedullary nail. We should not ignore the diameter of the femoral cavity by focusing only on the diameter of the intramedullary nail. In our study, the numbers of reoperation cases were as follows: 11 cases (13.7%) in high-matching group (gap ≤ 2 mm); 12 cases (13.8%) in middle-matching group (2 < gap < 4 mm); and 4 cases (11.1%) in low-matching group (gap ≥ 4 mm). No significant difference in reoperation rate was found among the three groups (*P* = 0.925, Table 2). Logistic regression showed no significant association was noted between matching degree of intramedullary nail and reoperation rate (Table 3).

The choice of intramedullary nail size remains controversial, which is often associated with complications resulting from the mismatch between the intramedullary nail and the femur, such as anterior cortical penetration and secondary fractures [15, 24]. Chang et al. found that impingement of the anterior femoral cortex occurred in 34.8% of the cases in a study involving 158 patients with intertrochanteric fractures treated with proximal femoral anti-rotatory intramedullary nail (PFNA) [25]. Although nailing with small- and large-diameter intramedullary nails had similar rates of fracture healing and secondary fracture [15], small-diameter intramedullary nails tended to be used to decrease the incidence of anterior cortical impingement [24, 26]; one scholar indicated that large-diameter nails would not bring more benefits to patients. Large-diameter nails relatively narrow the nail–canal gap, and the nail tip is more likely to “hit” the anterior femoral cortex and lead to perforated fractures [24]. It was reported other factors might lead to secondary fracture of the femur [27–29]. Secondary fractures are more likely to occur in patients with long nails [27]. The nail will be deformed when the long nail does not match the femoral marrow cavity because of differences in the radius of curvature, and deformation resistance will make the nail tip “hit” the anterior femoral cortex, resulting in perforated fractures [28]. For commercially available short nails, Ruecker et al. found that the bone cortex can be damaged by repeated drilling for distal locking or increased local stress, because the head of the distal locking bolt is too close to the lateral cortex, which may cause secondary femoral shaft fracture [29]. In our study, postoperative secondary femoral shaft fractures related to internal fixation were not observed, and the matching degree between proximal femoral intramedullary nail and femoral medullary cavity did not seem to be an important factor for reoperation, which supported that small-diameter nails could be used in lieu of large-diameter nails to reduce related complications.

In our research, the cutting of the screw was the most common failure pattern of internal fixation, with a total of 11 cases, all of which were accompanied by varying degrees of coxa varus. Differing from the report of Zhang et al., the most common failure mode was cutting-out of screw instead of cutting [11]. Cutting-out occurred in only two cases, because most patients were required to avoid weight bearing or removing the lag screw when cutting combined with coax varus was observed during the follow-up period. At a tip–apex distance of greater than 25 mm, screws placed in the unduly anterior or upper position and coxa varus were potential risk factors for the cutting of the screw [30].

Fracture reduction quality is one of the most important predictors to consider when preventing a second operation, because cortical buttress improves cortical resistance to collapse. Poor reduction, especially with head–neck fragment varus, increases the risk of cortical collapse [11]. Pressure is chiefly transmitted through internal fixation, which increases the risk of cutting bone with a helical blade [31]. These statements explain why the most common cause of reoperation in our paper was coxa varus combined with cutting (11 cases). According to the experience of Baumgaertner et al. [16], 103 cases (50.7%) were good, 67 cases (33.0%) were acceptable and 33 cases (16.3%) were poor in the reduction quality assessment. Logistic regression analysis (Table 3) showed that no strong dependency existed among explanatory variables, except for that between reduction quality and AO classification (6.798e−13). This finding demonstrated that unstable fracture patterns tended to lead to poor reduction quality. Unstable AO classification with poor reduction quality was the only significant variable where their 95% confidence interval does not include 1. Therefore, an association exists between unstable AO classification with poor reduction quality and reoperation (*P* = 0.002). There were 20 cases (9.85%) of OTA/AO 31A3 fractures, as follows: “Good” reduction grade in 0 case (0%); “Acceptable” in 7 cases (35%); and “Poor” in 13 cases (65%). The reduction quality of OTA/AO 31A3 fractures tended to be identified as “poor.” Six cases (30%) of OTA/AO 31A3 fractures required second surgery, and this reoperation rate was higher than in other studies [15]. OTA/AO 31A3 fractures lead to a high rate of internal fixation failure due to difficulty in reduction. Baumgaertner and Solberg et al. reported that poor reduction quality resulted in a threefold higher rate of internal fixation failure for posterior intertrochanteric fractures [32]. Hao et al. also demonstrated that poor reduction quality and defects in the posterior medial cortex are factors of internal fixation failure for posterior intertrochanteric fractures [33].

The effect of nail length on reoperation rate was analyzed. In our study, nails with lengths of 170, 200 and 240 mm were used. Logistic regression analysis showed that the length of nail was not significantly associated with reoperation (*P* > 0.05, Table 3), which was in accordance with findings of previous studies [34, 35]. The choice of the nail length was controversial. Although there were no significant differences in stability and failure of internal fixation between long and short nails reported in the current literature, patients on whom long nails were used experienced longer operation time and lost more blood [34]. Matching the long nail with femur was difficult due to the difference of the radius of curvature, thereby leading to a “hit” on the anterior femoral cortex and postoperative anterior knee pain, and surgeons prefer to use short nails [27].

## Conclusions

In summary, our studies found that the matching degree between proximal femoral intramedullary nail and femoral medullary cavity did not seem to be an important factor for reoperation, which offered more options of intramedullary nail size intraoperatively, reduced implants stock from inventory and costs to the healthcare systems and allowed orthopedic surgeons to used small-diameter nails in lieu of large-diameter ones for decreasing complications resulting from the mismatch between nail and bone.

### Limitations

This study has some deficiencies. Different surgeons operated on the patients. These surgeons received standardized training on the surgical approach, posture management, pre- and postoperative management. However, due to individual differences of surgeons, varying degrees of impact were bound to be made on pre- and postoperative management, fracture reduction quality, operative incision size, operative time, intraoperative posture and postoperative rehabilitation plans. Such differences affected the postoperative results, especially the reoperation rate. In addition, the diameter of femoral marrow cavity was not uniform, and thereby, the diameter was measured at the level where the medullary cavity is most fully filled with distal nail considering the feasibility of the study.

## Data Availability

The data are not publicly available due to their containing information that could compromise the privacy of research participants but are available from the corresponding author on reasonable request.
